# Participants’ experiences of living with cystic fibrosis related diabetes and using a hybrid closed-loop system to support self-management: qualitative study

**DOI:** 10.1186/s12902-026-02360-5

**Published:** 2026-07-07

**Authors:** Julia Lawton, Amanda I. Adler, Amanda Brennan, Richard I. G. Holt, Roman Hovorka, Nithya Kadiyala, Dawn Lau, Marian Peacock, Charlotte K. Boughton, David Rankin

**Affiliations:** 1 Usher Institute, School of Population Health Sciences, University of Edinburgh, Edinburgh, UK; 2 Diabetes Trials Unit, Oxford Centre for Diabetes, Endocrinology and Metabolism, University of Oxford, Oxford, UK; 3 Churchill Hospital, Oxford University Hospital NHS Foundation Trust, Oxford, UK; 4 Royal Papworth Hospital, Cambridge, UK; 5 Wolfson Diabetes and Endocrine Clinic, Cambridge University Hospitals NHS Foundation Trust, Cambridge, UK; 6 Manchester Adult Cystic Fibrosis Centre, Manchester University NHS Foundation Trust, Manchester, UK; 7 Human Development and Health, Faculty of Medicine, University of Southampton, Southampton, UK; 8 Institute of Metabolic Science-Metabolic Research Laboratories, University of Cambridge, Cambridge, UK; 9 All Wales Adult CF Centre, University Hospital Llandough, Wales, 2221 UK

**Keywords:** Cystic fibrosis-related diabetes, Cystic fibrosis diabetes, Cystic fibrosis, Hybrid closed-loop, Qualitative, Patient experience, Lived experience, Quality of life, Treatment burden, Self-management

## Abstract

**Background:**

Cystic fibrosis-related diabetes (CFrD) is a common complication of cystic fibrosis (CF). Many people with CFrD struggle to keep glucose within target range leading to poorer clinical outcomes and reduced life expectancy. Hybrid closed-loop (HCL) systems are an exciting innovation which may help people with CFrD improve their glucose management and experience a better quality-of-life. We explored the experiences of adults with CFrD who used the CamAPS FX HCL to: understand the benefits and challenges to using HCL in people with CFrD; and, help inform decision-making about HCL use in CFrD populations in routine clinical care.

**Methods:**

We interviewed 19 individuals who used the CamAPS FX HCL for ≥ 3 months during the CL4P-CF clinical trial. Data were analysed thematically.

**Results:**

Participants reported multiple clinical and quality-of-life benefits to using HCL. Many described treatment burnout before using the system and, hence, benefitting from automated glucose regulation and an increased capacity to take on new activities. Participants also shared historical difficulties gaining/maintaining weight as a result of their CF and described how using the system helped them achieve weight/muscle gain, improved their bodily confidence and enabled to them to enjoy a greater diversity of foods. Participants also observed CF-related benefits, including a (perceived) reduced susceptibility to infections, improved mood/energy to take on CF management-tasks, and increased confidence and ability to undertake physical activity due to a reduced risk of hypoglycaemia. In addition, participants reported medical trauma arising from hospital admissions in childhood and exposure to medically-invasive procedures for their CF, and highlighted benefits to using a system which mitigated the need to use injections. Due to their earlier traumatic experiences, some expressed a preference for using tubeless pumps and cannulas that require less frequent replacement.

**Conclusions:**

Participants reported improved glucose management, CF-related benefits and wide-ranging quality-of-life gains resulting from HCL use. Given their earlier experiences of treatment burnout and medically-related trauma, and taking account of the distinctive challenges involved in managing CFrD, our findings suggest that people with CFrD are a uniquely deserving group for access to HCL technology if shown to be clinically and cost effective in this population.

**Trial registration:**

ClinicalTrials.gov: NCT05562492, registration date 28th September 2022.

**Supplementary Information:**

The online version contains supplementary material available at https://doi.org/10.1186/s12902-026-02360-5.

## Introduction

Cystic fibrosis (CF) is a life-limiting condition, typically diagnosed soon after birth, which affects around 1 in every 2500 live births [1, 2]. It is caused by a gene mutation which results in viscous mucous secretions that affect multiple organs, including the lungs, pancreas, liver and intestines [3, 4]. Approximately 20% of adolescents and 40–50% of adults with CF develop CF-related diabetes (CFrD) [5]. The onset of CFrD is largely due to damage to the pancreas resulting from CF which leads to progressive insulin deficiency, although fluctuating levels of insulin resistance related to acute and chronic illness also play a role [3, 5–7]. The introduction of cystic fibrosis transmembrane conductance regulator (CFTR) modulator therapy, which has been widely used in routine care in the United Kingdom (UK) since 2019-20, has improved life expectancy for people with CF, resulting in more individuals surviving long enough to develop CFrD [8].

As a result of factors such as elevated airway glucose and impaired immune clearance, people with CFrD are more susceptible to respiratory infections, have poorer lung function and experience more pulmonary exacerbations than people with CF who do not have diabetes [5, 9–13]. In addition, hyperglycaemia trebles the risk of mortality in people with CFrD [14]. Hence, people with CFrD are advised to maintain glucose levels within clinically recommended ranges, typically by using multiple daily insulin regimens. To reduce the risk of insulin-induced hypoglycaemia, people with CFrD are also advised to check their glucose levels regularly, especially when sick, making dietary changes or undertaking physical activity. Use of carbohydrate counting to guide bolus insulin therapy is also encouraged [10, 15].

Given the dual demands of CFrD and CF management, many people with CFrD find it difficult to maintain glucose within target ranges, with studies highlighting low levels of engagement in self-management activities, such as glucose monitoring [16, 17], and high levels of treatment burnout [18, 19]. This has prompted decisions to explore the use of hybrid closed-loop (HCL) systems in people living with CFrD. Such systems automate insulin delivery via a continuous glucose monitoring (CGM) device and an algorithm-driven insulin pump. To date, several small-scale, short-duration studies have shown promising, albeit tentative, results, including improvements in HbA1c, glycaemic variability, treatment satisfaction and quality-of-life [20–23].

Larger-scale trials are now underway, including the Closed-loop for people living with Cystic Fibrosis related diabetes (CL4P-CF) trial (ClinicalTrials.gov identifier: NCT05562492, registration date 28th September 2022) [24], which is a UK-based, open-label, multi-centre, randomised, two-arm single-period parallel study comparing HCL with standard insulin therapy combined with CGM in people (≥ 16 years) with CFrD over a 6-month period. To complement the trial’s results, we conducted an embedded qualitative interview study, the findings from which we report here. The aim of this interview study was to understand and explore people’s experiences of using a HCL in their everyday lives, including any benefits and challenges arising from system use. Our objectives were to: address a vital gap in the literature as this is the first interview study to explore HCL use in people with CFrD; and, generate insights which could help inform decision-making about the rollout and use of this technology in people with CFrD in routine clinical care.

## Methods

We present our methods and findings broadly in line with the consolidated criteria for reporting qualitative studies (COREQ) [25], with Supplementary file 1 providing details for each of the 32 criteria.

### Overview

We interviewed adults with CFrD randomised to the HCL arm of the CL4P-CF trial. In this trial, participants used the CamAPS FX HCL system which comprises a Dexcom G6 CGM sensor (Dexcom, Northridge, CA, USA) usually attached to the arm, an YpsoPump insulin tubed pump (Ypsomed, Switzerland) which delivers insulin via an infusion set inserted under the skin usually on the abdomen, and a compatible smartphone. This system is operated via the CamAPS FX app (CamDiab, Cambridge, UK) hosted on the smartphone, which incorporates an adaptive algorithm that determines basal insulin needs and includes functions enabling users to input meal/snack bolus doses of insulin, personalise glucose targets and increase (‘Boost’) or reduce (‘Ease-off’) algorithm-driven insulin delivery. The app also facilitates automatic data upload to the Glooko cloud (Glooko, Göteborg, Sweden), enabling data-sharing with healthcare professionals.

Further details about the trial (including eligibility criteria) and CamAPS FX system are provided in Box 1. Ethics and governance approvals for the interview study were obtained as part of the main trial (REC ref: 22/EE/0164).

**Box 1:** Information about the trial and devices used

The CL4P-CF trial was conducted at 14 adult cystic fibrosis clinics across the UK [24]. To be eligible, participants had to be aged ≥ 16yrs, have lived with CFrD requiring insulin therapy for >3months, have a baseline time in glucose target range (4.0–10.0 mmol/l) of < 80% during the run-in period, a Forced Expiratory Volume (FEV_1_) > 30% of predicted mean for age, sex, race, and height at the screening visit or within the past 6 months and be willing to wear study devices. Participants were randomised to use either the CamAPS FX HCL (intervention arm) or standard insulin delivery with CGM (control arm) for 26 weeks. Participants were asked to contact their local study team for problems related to CFrD management, but also had access to a 24-h telephone helpline to contact the central study team for technical support.
CamAPS FX is a ‘hybrid’ closed-loop system calculating and delivering basal insulin automatically, but the user is advised to administer boluses to cover meals/snacks using the CamAPS FX app. The CamAPS FX app also includes functions enabling users to:
(1) view CGM glucose levels, glucose trend arrows and receive and personalise alarms (audio and vibration settings) triggered by high/low-glucose levels or system issues. (2) adjust algorithm-driven insulin delivery using a ‘Boost’ or ‘Ease-off’ mode of operation. (3) set personal glucose targets between 4.4 and 11.0 mmol/L (default is 5.8mmol/L)

### Recruitment

Participants were recruited into the interview study using an opt-in procedure when they were consented into the CL4P-CF trial. Recruitment took place at seven sites which were selected to ensure good geographical spread across the UK and to allow us to recruit a demographically mixed patient population. Purposive sampling was used to seek diversity with respect to individuals’ age, occupation, gender, CFrD duration and use of CFTR modulator therapy. However, in recognition that not all people can use or tolerate CFTR modulator therapy [26], we also represented non-CFTR users in our sample. Recruitment was stopped when data saturation was achieved (i.e., when no new findings were identified in new data collected).

### Data collection

We interviewed participants once after they had gained ≥ 3 months experience of using the HCL system. We chose this time-point to allow sufficient time for individuals to adjust to using the system and for system use to become embedded within their everyday lives [27, 28]. Interviews were conducted by a highly-experienced (non-clinical) qualitative researcher (DR, male) who was independent of the trial team. Interviews were informed by a topic guide (see Box 2), to ensure that areas explored were relevant to addressing study aims, while allowing participants opportunities to raise issues they considered important, including those unforeseen at the outset, in line with the flexible exploratory approach used in this study. The development of the topic guide was informed by reviews of relevant literatures (e.g., people’s experiences of living with and managing CFrD [16–18, 29], other user groups’ experiences of managing (type 1) diabetes using HCL [28, 30–34], input from clinical co-investigators and consultation work undertaken with people living with CFrD. As well as exploring participants’ experiences after moving onto HCL, we asked them to offer detailed accounts of what it was like for them to live with, and manage, their CFrD prior to using the system. This enabled us to tease out and understand the distinctive benefits (and challenges) they experienced as a result of HCL use. The topic guide was also revised in response to emergent findings. Specifically, we made a decision to also explore, in-depth, participants’ experiences of having CF in childhood and adolescence after our preliminary analysis of the first five interviews made it apparent that these historical, childhood experiences (e.g., of medical trauma resulting from lengthy hospital admissions and undergoing invasive medical procedures) could have a major impact on their subsequent experiences of using HCL, including the perceived benefits or challenges of doing so. Telephone interviews lasting 1–2 h took place between October 2023 and May 2025, were digitally-recorded and transcribed.

**Box 2:** Interview schedule

**Interviewee’s background and pre-trial experiences managing CF and CFrD**
1. Can you tell me a bit about yourself? (e.g., age, occupation, household composition). What do you enjoy doing in your free time? Are there any other health conditions you’re living with as well as managing CF/CFrD? 2. Establish if/when they started CFTR modulator therapy, whether they had received a lung transplant, and when they were diagnosed with CFrD – then adapt following questions depending on their responses. 3. When were you diagnosed with CF? [Pre-modulators] what’s it been like living with CF? [Probe: seek an account of the person’s life living with CF – how has it affected them, e.g., life choices/relationships; have periods of ill-health resulted in time off school/work; hospital admissions (for cold/flu, IV antibiotics); has it affected feelings of being ‘normal’; impact on childhood/adolescence and life goals]? 4. What have your experiences of needles been like when managing CF (e.g., for IV antibiotics, bloods being taken, etc.)? Any worries/concerns or anxieties? [Probe: childhood experiences]. 5. I understand that people with CF often struggle with gaining/maintaining weight, but could you tell me what your experience has been like? [Probe: thoughts about their appearance (weight and height), how they feel about their body, impact on confidence/self-esteem/relationships] 6. What, if any, difference has CFTR modulator therapy/lung transplantation made for you and your experiences of CF and CF management? [Probe: any differences in the kinds of things they feel able to do, weight gain/maintenance, how they think about and plan for the future]. 7. Tell me about your experiences when you were diagnosed with CFrD? What information and advice were you given about managing diabetes on top of CF? Has it affected your food choices; enjoyment of eating; confidence and ability to gain and maintain weight? 8. How did you find and approach doing injections (using needles), especially in light of your earlier experiences receiving medical care for CF (e.g., IVs, bloods taken on a regular basis)? 9. [If CFrD preceded modulators] After you’d begun to use modulator therapy (or post-transplant), did you think any differently about, or how you approached managing CFrD? Did this result in any changes in how you thought about your future and living with diabetes? Or changes in how you thought about the risk of complications arising from diabetes and what you might do to mitigate these? 10. What is the day-to-day reality like of having to manage two conditions – what sort of things do you have to do every day to manage both conditions? (e.g., medications, nebulisers, physio; diabetes – carbohydrate counting, insulin dose administration etc.) Are there occasions when you’ve had to prioritise one condition over the other? Has this changed over time? 11. Follow-up: Once you’ve done everything to manage both conditions, do you have time for other things in your life (relationships, hobbies, career)? Do you feel your life is very different to your peers? [Probe: reflections with others’ lives. Ask how the demands of other health conditions impact their day-to-day reality and workload, things they need to do to get by.] **Experiences of using closed-loop during the trial**
1. What were your perceptions and understandings of the closed-loop before you moved onto the trial? What were your reasons for taking part and how did you feel when you found out you had been randomised to the closed-loop arm? 2. How did you get on using the closed-loop and were there any challenges along the way? 3. How do you feel about taking a step back and letting the closed-loop operate versus stepping in and making diabetes-related decisions for yourself? 4. How did you feel about: wearing devices, impacts on feeling ‘normal’; feelings of being ‘ill’ because of using wearable technology? 5. Does your life look and feel any different now that you’ve been using the closed-loop for several months? For example, how do you feel about: • the things you have to do on a day-to-day basis to stay on top of (manage) both conditions? Do you feel you’ve had more or less work to do managing CFrD & CF while using the closed-loop? [Probe: What is it about the closed-loop that helps/hinders? ] • no longer having to inject insulin? • the kinds of things that you’re choosing or are able to eat; your enjoyment of eating and appetite [Probe: any changes to the kinds or amounts of food eaten, or time of eating; limits on ‘missing’ doses of insulin, e.g., for meals or snacks, compared with previous regimen]? • your experiences of weight gain/maintenance – have you noticed any differences? Relatedly, if benefits are noted, has using closed-loop affected your confidence/self-esteem in any way (if you’ve put on/maintained weight)? [Probe: how does closed-loop help/hinder? ] • doing physical activity – Has using a closed-loop affected, positively or negatively, what you’ve been able to do? Has this had any bearing/impact on your management of CF? 6. Do you feel like your management of CF has been affected by using a closed-loop? How has the closed-loop helped/hindered? 7. Has using the closed-loop affected how you feel about your quality of life in any ways we’ve not talked about? [Probe: ability/motivation to study/work; family/social life; opportunities for hobbies/physical activity; changes/improvements in sleep; physical health; mental health; impacts on others, e.g., partner/family; any changes in mood, confidence, self-esteem? ] 8. Has using a closed-loop had impacts on your health more generally? [Probe: feelings about their health, hospital admissions, developing ‘colds/flu’ and recovery following periods of ill-health] 9. Can you think of any ways in which continuing to use a closed-loop moving forwards might allow you to make new and different plans, or take on new activities, possibly different career or life choices, in the future? How would using closed-loop help? **Wrap-up questions**
1. Based on your experiences, is there any ways in which you think a closed-loop system might be improved to cater for people like yourself living with CF/CFrD? [Probe: use of cannulas and having to inject – if injections were/are an issue] 2. If we wanted to make a case to HCPs/funding bodies, how would we go about it – how can we make a case for people with CF/CFrD to be given better access to closed-loop? 3. Is there anything else you would like to add that we’ve not touched on?

### Data analysis

Interviews were analysed by two highly-experienced non-clinical, qualitative researchers (JL and DR) using a thematic approach [35], with additional input from MP who read the first seven interviews and contributed to topic guide refinement. Following completion of data collection, JL and DR read individual interview transcripts repeatedly (‘data immersion’) and then cross-compared accounts to identify initial, cross-cutting inductively generated themes, in line with the exploratory approach adopted in this study. Each researcher then wrote an independent report before meeting to discuss their interpretations and reach agreement on a coding framework which captured key analytical themes. Disagreement was minimal and resolved though discussion without need for third-party arbitration. Data were then coded to capture these themes and contextual information needed to aid data interpretation using the qualitative software package NVivo 20 (QSR International, Doncaster, Australia). Coded datasets were then examined in-depth to identify and refine themes and select illustrative quotations used in our reporting below.

## Results

Interviews were undertaken with 19 people living with CFrD. Two further people were approached but did not respond. See Table 1 for more information about the sample, including participants’ demographic characteristics. At the time of their interviews, five had received a bilateral lung transplant and 14 participants had been using CFTR modulator therapy for between 37 and 56 months.

**Table 1:** Sample characteristics, *n* = 19 participants with cystic fibrosis related diabetes

Characteristic	*n*	%*	Mean, SD, (range)
Male	10	52.6	
Age at time of interview; years			36.1 ± 9.2 (range: 21–51)
Married/co-habiting	12	63.2	
Employment			
Full-time Part-time Carer Not working due to health reasons	10 6 1 2	52.6 31.6 5.3 10.5	
Occupation^			
Managers Professionals Technicians and Associate Professionals Clerical Support Workers Skilled agricultural Service and Sales Workers Elementary Occupations (e.g., manual) Unemployed (due to health reasons) Full-time mother/carer	2 2 2 2 2 4 2 2 1	10.5 10.5 10.5 10.5 10.5 21.1 10.5 10.5 5.3	
Educational attainment			
GCSEs/O-levels (usually at age 15–16) A/AS-levels (usually at age 16–18) Other higher education University degree Postgraduate qualification or study	7 3 5 2 2	36.8 15.8 26.3 10.5 10.5	
Ethnicity			
White, British	19	100	
Using CFTR (modulator) therapy	14	73.7	
Use of CFTR modulator therapy; months			49.1 ± 6.1 (range: 37–56)
Received a lung transplant	5	26.3	
CF related diabetes (CFrD) duration; years			13.9 ± 7.3 (range: 1–29)
Insulin regimen used at baseline			
Basal/bolus Bolus only	15 4	78.9 21.1	
Baseline HbA1c			
mmol/mol %			74 ± 28 (range: 45–142) 8.9 ± 2.6 (range: 6.3–15.1)

* Figures may not add up to 100% due to rounding; ^ Defined using the International Standard Classification of Occupations 2008 (ISCO−08)

The analysis identified five overlapping analytical themes:’ Motivation and readiness to change’, ‘Treatment work’, ‘Body and eating’, ‘CF-related benefits’; and, ‘Trauma, injecting and tubing’. These are considered below. To protect participants’ identities, unique identifiers are used in our reporting. Unless otherwise indicated, findings did not differ according to participants’ gender and other demographic characteristics.

### Motivation and readiness to change

All participants described how, earlier in their lives, they had had to endure extremely punishing and time-consuming CF management regimens together with frequent, and often lengthy, hospital stays, (Table 2). They also reported growing up with the knowledge of having a short life-expectancy (Table 2) which, as some pointed out, had resulted in them not making plans for the future (Table 2). As participants further pointed out, these factors in combination often led to them neglecting CFrD management tasks, such as administering insulin regularly and undertaking frequent glucose checks, with 003 noting how:

**Table 2:** Supplementary quotations

THEMES	Sub-themes and additional illustrative quotations
Motivation and readiness to change	*Punishing and time-consuming CF management and lengthy hospital admissions:* “So medication wise, I was on probably ten different drugs at breakfast time…Creon throughout the day, with anything I ate, that was more complex than just sugar, another six tablets in the evening. I was doing two nebulizers a day of antibiotic, I was needing to try and exercise fairly regularly, so I’d try and get out for a walk a couple of times a day, or get out on my bike, or do some kind of HIT training in the house, like something along those lines. And that’s, yeah, I found that quite a struggle to do that kind of stuff. But I realised it’s quite important for my chest.” (018) “it’s awkward…you’re pretty much sorting out all your medications, making sure that you’ve done your physio. I mean it’s quite gruelling, and…demanding…I ended up with chest infections…and then I’d be in hospital for a month…it’s awkward trying to have a normal life, because…you’ve got to know your limitations with it.” (019) “I’d have IV antibiotics [as an in-patient], for two weeks, or three weeks sometimes. And then, as soon as they stopped, I would spend a couple of weeks of being okay, and then I would just deteriorate again. And then I’d follow that cycle through and over and over again really.” (007) *Views about life expectancy:* “When I was a kid, they told my mum that I wouldn’t make it into teenage years…And then when I made it to teenage years it was like, well, she unfortunately won’t make it into early adulthood.” (015) *Not making plans for the future:* “it just goes hand in hand [having CF and not forming relationships] not approaching people in bars…if me and this person got together, well, I’m going to be gone in a few years, so that’s just mean to them. And I never had confidence to do that kind of thing, and I feel a lot in my 20s I just drifted by and not actually took opportunities like that.” (016) *Improvements in lung function when using modulator therapy:* “when I started it [modulator therapy] I just couldn’t believe the change within days of being on it basically…my lung function went from 35 up to like 45 [% FEV_1_]. I’m between 70 and 72 [% FEV_1_], and that’s four years down the road. So it has changed my lung function massively.” (004) *CFrD viewed as a more pressing issue when using modulators or after lung transplant:* “I think obviously when I did start my Kaftrio, and over time when like it got into my system, and I really saw like more of the benefits, and the CF symptoms sort of subsiding a bit, it did sort of make me think: well okay, obviously I’ve still got diabetes, that’s not going away, cause my pancreas doesn’t work. It did make me think: okay, well my main focus now needs to be my diabetes.” (014) “At the same time as I getting used to the transplant, it kind of threw a different perspective on things, with everything kind of stabilizing, it very much felt like diabetes became more of a problem because the other issues [CF management] became less of a problem.” (018) *CF can impact glucose levels in unpredictable ways:* “But the CF side of it also complicates the diabetes side of it, because as soon as you get lung infections, or chest infections, it just makes your diabetes ten times worse. So really hard to control it and you have to learn to adjust your own dosage of insulin whereas a normal diabetic doesn’t do that really, a Type 1, they’re more static in terms of their dosage, and their regime, and they don’t get these peaks and troughs and variability that CF diabetes patients do really.” (007)
Treatment work	*Managing CF and CFrD results in physical and mental exhaustion:* “The average person just has to eat, whereas a person with CF and CFrD, before I eat something, I’m thinking: okay how much fat has it got, how much Creon do I need for that? And then I think: how many carbs, how much do I need for that…And you’ve got to be consciously planning ahead - if I’m going to work, have I got all my tablets with me…I’ve got to pack my insulin…that constant thinking about yourself…I was just exhausted.” (002) *Treatment burden (for those with multiple co-morbidities):* “So I have CF related diabetes. I’ve got CF. I have Lynch syndrome, hypertension, got CF related liver disease, CF related sinus disease, asthma. I have kidney stone issues. I have sleep apnoea, reflux. I have an overactive bladder now, high cholesterol, and recently been diagnosed with Lupus, and rheumatoid arthritis… loads going on…[it’s] just huge, yeah. So medication wise is just phenomenal to be honest, to manage.” (007)
Body and eating	*Reluctance to snack:* “especially snacking, because whereas before if I was to have a snack I would just take…a couple of Creon…Sometimes like really early into my [diabetes] diagnosis I would think: ugh, is it worth getting a needle out and jabbing myself, just for…a few Jaffa cakes or whatever…so, instead of eating the Jaffa cakes and not doing the insulin, I just didn’t eat the Jaffa cakes.” (016) *Avoiding eating energy dense meals:* “But it’s when you start going into the higher carbohydrate territory, a hundred carbs plus per meal, which I know it sounds a lot, but I’d be eating stuff like that sometimes…it doesn’t scale up as well. I feel like…if I’m eating something that I know’s got say 120 grammes of carbs in it…you can easily get that if you eat a McDonald’s can’t you, with a milk shake, it’s easily achieved. So I find that would be six units and that would almost be too much. That would send me into a hypo.” (017) *Weight and muscle gain from using closed-loop:* “I’ve been training properly again, and I must have gained 12 kilos in the last three weeks. So it’s putting on weight very quickly this time. It normally takes me about a month to put 12 kilos on and it’s done it within two, three weeks.” (019) …due to the system’s ability to regulate and optimise blood glucose levels: “So when I’m doing heavy weight training and stuff, you have to couple that with good sleep, cause that’s when your body recovers, and you grow. And if you’re out of range while you’re sleeping…your repair and growth and recovery isn’t optimised if it’s out of range. So being on the closed-loop, I think, allows me to get the most out my work-outs, because I know that I’m gonna stay in range all night, and that means that the glucose is fuelling my cells.” (010) *More confident and able to eat a diversity of foods:* “it gives me more confidence to (exhales) to not need to worry so much about what I’m eating. And by that I mean if I’m out at a friend’s house, and there’s stuff to snack on, or there’s, I dunno, maybe pineapple out on the table as a dessert, and I don’t know what the carbs are in pineapple. I know what they are in apple, I know what they are in banana, because I have those regularly at home. I don’t have pineapple regularly at home, is that I have a lot more confidence to eat those foods, knowing that the closed-loop system will make those corrections for me. So, yes, there’s definitely an improved ability to be more flexible with my food choices.” (018)
Benefits to CF	*Improvements in glucose management help strengthen the immune system:* “So we all know with higher blood sugar, that’s festering for germs and bacteria. So your blood gets really syrupy, and you harbour more infections. Your body, your lungs, it’ll grow and grow, it’s the perfect sort of environment for these bacterias and germs to reproduce. So, the better our blood sugar is controlled, the fewer infections we have. Like that’s a fact. When it’s controlled with the closed-loop system, or insulin pen, it doesn’t matter. But with the closed[-loop] system it’s so much easier to do, and so much better to do.” (013) *Wider economic benefits:* “But I think the money it saves, like the less I need to go and see my [healthcare] team, the less I need to go to A&E, because I have got something wrong with me, the better I keep because of a better controlled blood sugar, fewer infections, better mental health, better physical health. That has to count, like we have to realise how many fewer hospital visits we need, how much more I can contribute to society with my job, how many more hours I can work, how much more of a valuable asset I am to my community. I think that is also monetary.” (008) *Improvements in mood and energy have benefits for CF management:* “…whenever my sugar levels were really high, I’d just feel like really bleugh, like lethargic and stuff. So I would always find it more like trickier to do my physio, do my exercise. And then also on the other end of the spectrum, if I was having hypos, I really couldn’t do anything…I’d just feel too wiped out to even think about like doing my medications… but now - I think the main thing is that it does just free up a bit of time, and a bit of like mental capacity to then think about my CF.” (014) *Fewer hypos increases confidence to undertake other activities:* “it allows me to do things which are good for my chest, that I often wouldn’t be able to do before the closed-loop system because my sugars weren’t in a place to allow it. So for example, [before using closed-loop] I would maybe have a window between four and six o’clock in the evening where I could get out for a walk, and if the app was showing my blood sugars were [going] down at 4.2 [mmol/L], then I’d have to abandon that, so I’d lost my window of opportunity.” (018)
Trauma, injecting and tubing	*Needle phobia:* “Because when I’d go into hospital, they’d put in a long line which involves getting a needle…and the doctors would always put the needle in first and then dig around and try and find a vein. It really threw me as a kid into not liking any kind of injection.” (006) …which had an adverse impact on glucose management “I have quite a needle phobia which meant my diabetes management took quite a downfall…My bloods keep rising and rising because I wasn’t doing the finger pricks as much as I should have been and I wasn’t doing corrections.” (005) *Closed-loop improvements: less frequent replacement of the cannula:* “So we have to change the cannula every two, to maximum three days…if that could be improved to maybe changing it once a week, that could be massive, cause then it’s only one injection once a week, as opposed to a possible two or three.” (006) …and use of a tubeless pump: “It’s an area where I think it needs to be improved, I think, the long cable [tube], having it come out, it triggered me a little bit.” (010)

I was just at that point in life where I thought what’s the point, it’s not gonna do anything. And I missed injections, I totally didn’t do them at all…I just thought, I’ve got CF, so why bother? (003)

After starting modulator therapy or receiving a lung transplant, participants reported improvements in their lung functioning (Table 2), which not only resulted in them having more energy to focus on their CFrD but also to them now seeing their CFrD as being a more pressing and serious health issue than their CF (Table 2). Additionally, participants, especially those using modulator therapy, reported an increased motivation to improve their CFrD management because improvements in their life-expectancy meant that they now had to be “mindful of some of the long-term risks…things like my cardiovascular health are a lot more important now.” (006).

Participants, however, described struggling to translate their increased motivation into tangible benefits, due to the extreme difficulties they experienced keeping their glucose within clinically-recommended target ranges:

There was no rhyme or reason to the high glucose readings you’re getting when you’ve injected double what you would normally do, and you know, or you’ve not even eaten for hours, and- and yet your sugars are going through the roof sort of thing…it drives you nuts to be honest. (003)

Participants often attributed these difficulties to having CF because, as they observed, this condition could affect their blood glucose in sudden and unpredictable ways (Table 2). Consequently, many described having become “desperate” (009) and, hence, being very receptive to giving “the algorithm a go” (009) when they were approached about the trial, because:

You start looking into the future and thinking…My lung function is good. I’m fit and healthy in lots of different ways…But the one thing that remained a niggle, I knew the diabetes needed attention as I was really struggling with it, and I thought this [chance to use closed-loop] was a great opportunity. (002)

### Treatment work

Despite seeing use of modulator therapy or a lung transplant as a watershed moment, many participants acknowledged that their lives had continued to be affected by lethargy and episodes of (extreme) fatigue, wherein: “sometimes I’ll have like two or three weeks where I’m absolutely wrecked” (019). In addition, participants noted how, while they now needed to expend less time and effort managing their CF, staying on top of this condition continued to entail treatment work because they still needed to take tablets (e.g., Creon with every meal/snack, immunosuppressants for those who had had lung transplants), with most also describing on-going use of nebulizers and physiotherapy. As a consequence, many described how the cumulative demands of managing both CF and CFrD had resulted in physical and mental exhaustion in the years leading up to the trial (Table 2), with such participants alluding to treatment burnout, because “managing our health is at least a part-time job” (008). This issue was expressed even more keenly by those who, by virtue of having multi-morbidities (e.g., gastro-oesophageal reflux disease, distal intestinal obstruction syndrome, CF-related liver disease, hypertension, sinusitis, anxiety, depression, asthma and arthritis), were on additional, and often highly complex, treatment regimens (Table 2).

After moving onto the system, participants typically reported feeling “relieved, to be honest” (019) because the system’s ability to automatically regulate their glucose and help keep it within target range meant they now needed to undertake less CFrD treatment work:

it’s just taken the burden of diabetes off the plate. I don’t have to worry about injecting, or there’ll be times where I’ve gone out in the past and forgotten my pen…Whereas this is actually attached to me, I can’t forget that (laughs) I think just in general, it’s made it very, very easy. (003)

Indeed, many described how, for the first time in lives, they had felt able to take on new hobbies and activities, with 018 noting how she was now leading a fuller life without hitherto having realised that crucial elements had been missing:

I didn’t realise how much of my time it took up, it was just normal to me, I had no idea what it was like not to do those things…So it’s been really freeing to have the headspace and the ability just to be impulsive and do things without having to pre-plan. (018)

### Body and eating

Many participants reported how, historically, their CF had led to them having a complex, and often highly negative relationship with food. Specifically, participants described how earlier recommendations to eat a very high calorie diet had undermined their enjoyment of eating because, “my whole life has been eat as much as you can all the time and it meant that food became much more of a medication than a pleasure” (018). 008 in a more extreme example still recounted how:

Eating itself became a chore…because I didn’t have much appetite cause I was coughing all the time. I was vomiting sometimes through the coughing fits…and also not having the air to breathe, so I was losing weight very quickly. (008)

Many described struggling to gain and maintain weight as a consequence, which had had very detrimental impacts on their confidence and self-esteem, due to “looking like a beanpole” (002) and/or them worrying that other people did not find them attractive:

He [partner] thought he was doing a nice kind thing telling me I don’t need to lose all that weight and he doesn’t like me thin…and you know, not having breasts, not having any sort of figure really, really affected me…my confidence took a massive hit. (004)

Many also noted how their relationship with their own bodies and food had been further complicated by having CFrD. Some, for instance, reported a reluctance to snack in order to avoid having to inject insulin, which could compound their problems with weight loss/maintenance (Table 2). Others described having avoided eating high carbohydrate, energy dense meals because of the difficulties they had encountered determining the carbohydrate content accurately and, hence, the high likelihood of administering too much insulin and then experiencing hypoglycaemia (Table 2).

While using modulator therapy or having a lung transplant enabled many participants to achieve weight gain before joining the trial, some also described the HCL as conferring additional, positive benefits. Such participants, typically men, reported being able to attain rapid weight and muscle gain, especially when using the HCL in conjunction with weight/resistance training (Table 2), because of the system’s ability to help regulate and optimise their blood glucose levels (Table 2). These participants also reported positive impacts on their confidence and self-esteem because as, as 010 explained:

I’ve gone through my whole life in being really underweight, really small…and…my struggles with CF have massively moulded who I am as a person…I’ve always wanted to be bigger and stronger…I wanted to get a picture alongside my friends and not be the smallest one there…and it’s- you know, gaining this weight has done so much for my confidence. (010)

Others noted how, after transitioning onto the system, they had felt more confident and able to eat a wider diversity of foods, due to not having to inject insulin and the system’s ability to automatically correct high/low glucose (Table 2), which as 012 noted, “makes me feel happier, it’s like a freedom.”

### CF-related benefits

Participants often reported a long history of battling viral and bacterial infections which had often involved lengthy (2 + weeks) hospital admissions and had worsened their lung functioning:

I was very ill, all the time really, with infections on infections, and I was being admitted to hospital every 4–6 weeks for IV treatment. I was in and out of hospital a lot…I was getting to a scary place. (007)

While many highlighted benefits to using modulator therapy or having had a lung transplant, participants also observed a further reduced susceptibility to infections after transitioning onto the HCL and ensuing improvements to their quality-of-life. Although they often struggled to provide scientific explanations, some attributed this to the weight gain they had achieved as a result of using the system: “if I’ve got the weight behind me it helps with chest infections” (004). Others suggested that improvements in their glucose management had strengthened their immune systems (Table 2) and improved their ability to clear bacterial infections without antibiotics, because “[if glucose is more in-range] it makes the sputum less sticky, which is [then] easier to get out” (012). When they offered these kinds of reflections, many were keen to emphasize that using the system had not only conferred personal CF-related health benefits but also had potential to offer wider economic benefits due to users needing less time off work and fewer hospital admissions (Table 2).

After moving onto HCL, participants also reported improvements in their mood and energy levels, which had resulted in them being more likely to undertake breathing exercises and/or use nebulizers to optimally manage their CF (Table 2). Some further reflected on how reduced occurrences of hypoglycaemia resulting from system use had increased their confidence and ability to undertake activities such as walking and running (Table 2), which “is good for my lung health” (009).

### Trauma, injecting and tubing

When they recounted their earlier experiences of lengthy, repeated hospital admissions, especially those which had occurred in childhood and adolescence, most participants alluded to medically-related trauma. This trauma, as participants’ accounts suggested, had arisen not only from experiencing episodes of extreme sickness, but also from having had to endure painful, invasive medical procedures (e.g., repeated blood tests, insertion of Port-a-Caths and/or a percutaneous endoscopic gastrostomy (PEG) feeding tube) throughout their formative years:

I remember especially the PEG, because the PEG putting-in was an issue, because they did it under local anaesthetic, so they had to put a tube down my throat, which when you can’t breathe, it is an issue…And the Port-a-Cath as well, they realised on one side my veins are smaller, so that couldn’t be put in on that side. So I remember lying there in hospital thinking: Christ. (011)

For some, these childhood experiences had led to them developing a needle phobia (Table 2), which had had adverse impacts on their glucose management pre-HCL (Table 2). Others, including 010, expressed an extreme dislike of seeing or having tubing attached to their bodies due to childhood experiences of having had to use peripherally inserted central catheter (PICC) lines and/or PEG:

I struggled, because I’ve got quite a lot of trauma from previous treatments…I’ve been on intravenous antibiotics and all that. And when they got this pump out in front of me, and you’ve got the really long blue tube, it put me in a really weird- it almost put me back to how I used to feel, just seeing the tube, that would then be coming out of me, you know, it brought up some really bad feelings. (010)

As a consequence of these earlier, highly negative treatment encounters, many described appreciating using a technology which enabled insulin to be administered without having to inject or undertake regular finger-prick checks. Some, however, also indicated that their HCL experiences could by improved by using an infusion set (including cannula) which requires less frequent replacement (Table 2) and/or a tubeless pump (Table 2).

## Discussion

This interview study explored participants’ experiences of using HCL to support their CFrD management. In doing so, we have identified multiple benefits to system use, many of which were influenced by the distinctive challenges presented by living with both CF and CFrD and earlier, negative CF-related treatment experiences. Many participants described treatment burnout and exhaustion prior to using HCL and, hence, benefitting from automatic glucose regulation and an increased capacity to take on new activities. Participants also shared historical difficulties gaining and/or maintaining weight as a result of their CF and described how system use helped them achieve weight and muscle gain, improved their bodily confidence and self-esteem and enabled to them enjoy a greater diversity of foods. Participants also observed CF-related benefits, including a perceived reduction in their susceptibility to infections, improved mood and energy which helped enable them to take on CF management-tasks, and an enhanced confidence and ability to undertake physical activity due to a reduced risk of hypoglycaemia. In addition, participants reported medical trauma resulting from earlier hospital admissions and exposure to medically-invasive procedures for their CF, and highlighted benefits to using a system which mitigated the requirement to use injections. Due to their earlier traumatic experiences, some expressed a preference for using tubeless pumps and cannulas requiring infrequent replacement.

Many of the benefits participants reported have also been highlighted by other system users, principally those with type 1 diabetes amongst whom most HCL research has been conducted. A widely reported benefit, for instance, is the system’s ability to automatically regulate glucose, with resultant reductions in people’s diabetes treatment work [28, 32, 36]. Similarly, studies have found that HCL users appreciate the increased dietary permissiveness which system use affords, including opportunities to consume larger, high-carbohydrate meals and to snack without needing to inject insulin [30, 37, 38]. Additionally, HCL users report appreciating being able to administer insulin via a pump rather than having to use painful/visible injections [28, 33].

However, given the distinctive, highly negative historical experiences participants in our own and other studies have described, these kinds of benefits may be felt particularly keenly by those living with CF and CFrD. Many participants, for instance, reported treatment burnout prior to using HCL, due to the dual demands of managing CF and CFrD [18, 19] alongside other co-morbidities. Indeed, in line with others’ observations [39–41], participants reported continuing to feel burdened by CF-related treatment work even after introduction of modulator therapy or a lung transplant. Additionally, maintaining glucose within target ranges can be particularly difficult for people with CFrD, due to the disease’s distinctive pathophysiology which is characterised by insulin deficiency and intermittent insulin resistance (from infections and corticosteroid therapy), exocrine pancreatic insufficiency as well as people’s high-carbohydrate nutritional needs [7, 10, 42]. As our findings suggest, treatment burnout not only influenced participants’ receptiveness to using HCL, but also their relief after being able to delegate CFrD treatment work to the system. HCL receptiveness also appeared to be informed by the shifting CF treatment landscape. Specifically, as we and others have shown, improvements in life-expectancy and reductions in CF cognitive burden following introduction of modulator therapy or lung transplantation can result in individuals having a more future-oriented outlook [29, 43, 44] and to them now wanting and being able to prioritise their CFrD [29, 44].

Consistent with our observations, other studies have found that disordered eating and body dissatisfaction are common among people with CF [17, 45], with males being especially vulnerable to bodily dissatisfaction due to delayed puberty [46] and representations of masculinity conflicting with the low body weight resulting from CF and CFrD [17]. While modulator therapy has helped address long-standing challenges gaining and maintaining weight [44], some of our participants, especially male gym users, described being better able to gain/maintain weight and muscle-mass while using HCL; an effect which may be due in part to reduced catabolic hyperglycaemia and the anabolic effect of insulin itself. Additionally, some participants noted how, by lowering their risk of hypoglycaemia, system use enhanced their confidence and ability to undertake physical activities such as walking and running which had had positive impacts on their CF management. These observations find some support in the literature, with studies suggesting that resistance training and physical exercise interventions can help improve respiratory muscle functioning as well as health-related quality-of-life more generally [47, 48]. The potential for HCL use to optimize the benefits of these kinds of interventions, and to encourage people to follow exercise and physical activity guidelines for people with CF [49] therefore warrants serious consideration.

Participants reported experiencing a reduced susceptibility to lung infections and suggested that this was a key benefit to HCL use which was particularly meaningful to themselves. Although they often struggled to provide scientific explanations, some studies support our participants’ observations by highlighting associations between reductions in hyperglycaemia, pulmonary exacerbations and lung infections [7, 10, 11]. Given these findings, it may be important to ensure measures of infection frequency, hospital admissions and episodes of sick-leave are included in trials and other studies exploring HCL use in CFrD populations to allow the wider (potential) health economic benefits to be captured, in line with our participants’ suggestions.

Like others, many participants described experiences of medical-traumatic distress [50], arising from repeated, painful and highly invasive medical procedures for their CF during childhood and adolescence. Indeed, as others have similarly found, needle-related anxiety is very common among people with CF [51]. While prior studies have highlighted detrimental impacts on CF management [50–52], our findings suggest that medical-traumatic distress can also result in diabetes-related tasks (e.g., insulin administration) being neglected in those who have CFrD. Given the kinds of trauma our participants shared, it is understandable that many appreciated being able to use a system which removed the requirement to inject insulin. Indeed, if people with CFrD are unable to access HCL in routine clinical care, they might still benefit from opportunities to use insulin pumps to support optimal glucose management and enhance quality-of-life. As our findings further illustrate, where possible, individuals should also be given opportunities to use tubeless pumps and infusion sets requiring less frequent replacement, whether as part of an HCL or as standalone devices.

A key study strength is our use of a flexible, open-ended design, along with our decision to explore historical (pre-HCL) experiences, as this provided valuable, contextual information which enhanced understanding of HCL benefits within this particular population. The interviews were conducted by an interviewer who was independent of the trial team which helped create opportunities for participants to disclose negative experiences. We were successful in recruiting a demographically diverse sample across multiple trial sites, which increases the generalizability of our findings. However, participants were recruited via a trial and likely received more contact and support than would happen in routine clinical care. This may have resulted in them sharing particularly positive experiences. We undertook long, detailed interviews which also explored participants’ lived and self-management experiences prior to using HCL. While this approach allowed us to identify and explore the distinctive benefits (and challenges) which can result from using HCL, participants’ historical experiences may have been subject to recall bias. Hence, researchers may wish to consider using longitudinal (pre- and post-HCL use) designs in future studies, although this would increase the time and costings associated with doing the research. Additionally, participants only used one type of HCL. Hence, future research could involve users of different systems, including those in routine clinical care. Finally, while our sample included some people who were not using modulator therapy, these individuals had all benefited from lung transplantation; hence, we may have missed participants who might have particularly appreciated using HCL (i.e., those not using modulator therapy and who had not had a lung transplant). Indeed, as others have observed, individuals who have CFTR genotypes which make them unresponsive to current modulators or have experienced significant side-effects necessitating therapy cessation are particularly vulnerable to feeling demotivated, abandoned, and scared [44].

## Conclusions

This study is the first to explore experiences of HCL use among people with CFrD. Participants reported experiencing improved glucose management, CF-related benefits and wide-ranging quality-of-life gains resulting from HCL use. Given their earlier experiences of treatment burnout and medically-related trauma, and taking account of the distinctive challenges involved in managing CFrD, our findings suggest that people with CFrD are a uniquely deserving group for access to HCL technology if shown to be clinically and cost effective in this population.

## Electronic Supplementary Material

Below is the link to the electronic supplementary material.

Supplementary Material 1

## Data Availability

The datasets generated and/or analysed during the current study are not publicly available because, even after removal of identifying information (e.g., names and locations) from the interview transcripts, it may still be possible for some individuals, such as health professionals who provide clinical services, to identify the people who took part in the study. This is because of the small number of people who participated and because they provided detailed accounts about living with and managing CFrD in the context of their everyday lives. Raw data are available from the corresponding author on reasonable request.

## Abbreviations

CF: Cystic fibrosis
CFrD: Cystic fibrosis related diabetes
CFTR: Cystic Fibrosis Transmembrane Conductance Regulator
HCL: Hybrid closed-loop
CGM: Continuous glucose monitor
FEV_1_: Forced Expiratory Volume
UK: United Kingdom
