# Supplementary material for: Participants’ experiences of living with cystic fibrosis related diabetes and using a hybrid closed-loop system to support self-management: qualitative study
Source: BMC Endocr Disord. 2026 Jul 7;26:297. doi: 10.1186/s12902-026-02360-5 (PMC13628715; doi:10.1186/s12902-026-02360-5)
Supplement: Supplementary file 1 [file 12902_2026_2360_MOESM1_ESM.docx]

**Box_2. Interview topic guide**

**Interviewee’s background and pre-trial experiences managing CF and CFrD**

1. Can you tell me a bit about yourself? (e.g., age, occupation, household composition). What do you enjoy doing in your free time? Are there any other health conditions you’re living with as well as managing CF/CFrD?
2. Establish if/when they started CFTR modulator therapy, whether they had received a lung transplant, and when they were diagnosed with CFrD – then adapt following questions depending on their responses.
3. When were you diagnosed with CF? [Pre-modulators] What’s it been like living with CF? [Probe: seek an account of the person’s life living with CF – how has it affected them, e.g., life choices/relationships; have periods of ill-health resulted in time off school/work; hospital admissions (for cold/flu, IV antibiotics); has it affected feelings of being ‘normal’]?
4. What have your experiences of needles been like when managing CF (e.g., for IV antibiotics, bloods being taken, etc.)? Any worries/concerns or anxieties? [Probe: childhood experiences].
5. I understand that people with CF often struggle with gaining/maintaining weight, but could you tell me what that’s been like in your experience? [Probe: thoughts about their appearance (weight and height), how they feel about their body, impact on confidence/self-esteem]
6. What, if any, difference has CFTR modulator therapy made for you and your experiences of CF and CF management? [Probe: any differences in the kinds of things they feel able to do, weight gain/maintenance, how they think about and plan for the future].
7. Tell me about your experiences when you were diagnosed with CFrD? What information and advice were you given about managing diabetes on top of CF? Has it affected your food choices; enjoyment of eating and ability to gain and maintain weight?
8. How did you find and approach doing injections (using needles), especially in light of your experiences receiving medical care for CF (e.g., IVs, bloods taken on a regular basis)?
9. [If CFrD preceded modulators] After you’d begun to use modulator therapy (or post-transplant), did you think any differently about, or how you approached managing CFrD? Did this result in any changes in how you thought about your future and living with diabetes? Or changes in how you thought about the risk of complications arising from diabetes and what you might do to mitigate these?
10. What is the day-to-day reality like of having to manage two conditions – what sort of things do you have to do every day to manage both conditions? (e.g., medications, nebulisers, physio; diabetes – carbohydrate counting, insulin dose administration etc.) Are there occasions when you’ve had to prioritise one condition over the other?
11. Follow-up: Once you’ve done everything to manage both conditions, do you have time for other things in your life (relationships, hobbies, career)? Do you feel your life is very different to your peers? [Probe: reflections with others’ lives. Ask how the demands of other health conditions impact their day-to-day reality and workload, things they need to do to get by.]

**Experiences of using closed-loop during the trial**

1. What were your perceptions and understandings of the closed-loop before you moved onto the trial? What were your reasons for taking part and how did you feel when you had been randomised to the closed-loop arm?
2. How did you get on using the closed-loop and were there any challenges along the way?
3. How do you feel about taking a step back and letting the closed-loop operate versus stepping in and making diabetes-related decisions for yourself?
4. How did you feel about: wearing devices, impacts on feeling ‘normal’, visibility or other manifestation of having CFrD; feelings of being ‘ill’ because of using wearable technology?
5. Does your life look and feel any different now that you’ve been using the closed-loop for several months? For example, how do you feel about:

- the things you have to do on a day-to-day basis to stay on top of (manage) both conditions? Do you feel you’ve had more or less work to do managing CFrD & CF while using the closed-loop? [Probe: What is it about the closed-loop that helps/hinders?]
- no longer having to inject insulin?
- the kinds of things that you’re choosing or are able to eat; your enjoyment of eating and appetite [Probe: any changes to the kinds or amounts of food eaten, or time of eating; limits on ‘missing’ doses of insulin, e.g., for meals or snacks, compared with previous regimen]?
- your experiences of weight gain/maintenance – have you noticed any differences? Relatedly, if benefits are noted, has using closed-loop affected your confidence/self-esteem in any way (if you’ve put on/maintained weight)? [Probe: how does closed-loop help/hinder?]
- doing physical activity – Has using a closed-loop affected, positively or negatively, what you’ve been able to do? Has this had any bearing/impact on your management of CF?

6. Do you feel like your management of CF has been affected by using a closed-loop? How is it that the closed-loop helps/hinders?
7. Has using the closed-loop affected how you feel about your quality of life in any ways we’ve not talked about? [Probe: ability/motivation to study/work; family/social life; opportunities for hobbies/physical activity; changes/improvements in sleep; physical health; mental health; impacts on others, e.g., partner/family; any changes in mood, confidence, self-esteem?]
8. Has using a closed-loop had impacts on your health more generally? [Probe: feelings about their health, hospital admissions, developing ‘colds/flu’ and recovery following periods of ill-health]
9. Can you think of any ways in which continuing to use a closed-loop moving forwards might allow you to make new and different plans, or take on new activities, possibly different career or life choices, in the future? How would using closed-loop help?

**Wrap-up questions**

1. Based on your experiences, is there any ways in which you think a closed-loop system might be improved to cater for people like yourself living with CF/CFrD? [Probe: use of cannulas and having to inject – if injections were/are an issue]
2. If we wanted to make a case to HCPs/funding bodies, how would we go about it – how can we make a case for people with CF/CFrD to be given better access to closed-loop?
3. Is there anything else you would like to add that we’ve not touched on?
